# WGEVIA: A Graph Level Embedding Method for Microcircuit Data

**DOI:** 10.3389/fncom.2020.603765

**Published:** 2021-01-06

**Authors:** Xiaomin Wu, Shuvra S. Bhattacharyya, Rong Chen

**Affiliations:** ^1^Department of Electrical and Computer Engineering, University of Maryland, College Park, MD, United States; ^2^Department of Diagnostic Radiology and Nuclear Medicine, University of Maryland School of Medicine, Baltimore, MD, United States; ^3^Institute for Advanced Computer Studies (UMIACS), University of Maryland, College Park, MD, United States

**Keywords:** graph embedding, machine learning, neural decoding, microcircuit analysis, calcium imaging

## Abstract

Functional microcircuits are useful for studying interactions among neural dynamics of neighboring neurons during cognition and emotion. A functional microcircuit is a group of neurons that are spatially close, and that exhibit synchronized neural activities. For computational analysis, functional microcircuits are represented by graphs, which pose special challenges when applied as input to machine learning algorithms. Graph embedding, which involves the conversion of graph data into low dimensional vector spaces, is a general method for addressing these challenges. In this paper, we discuss limitations of conventional graph embedding methods that make them ill-suited to the study of functional microcircuits. We then develop a novel graph embedding framework, called Weighted Graph Embedding with Vertex Identity Awareness (WGEVIA), that overcomes these limitations. Additionally, we introduce a dataset, called the five vertices dataset, that helps in assessing how well graph embedding methods are suited to functional microcircuit analysis. We demonstrate the utility of WGEVIA through extensive experiments involving real and simulated microcircuit data.

## 1. Introduction

Graph-related data is widely used in real world applications, including social network graphs and customers' interest graphs in consumer applications (Huang et al., 2004), molecule and protein networks in biology and chemistry (Yue et al., 2020), and brain networks (Chen et al., 2017) for neuroscience and biomedical engineering. Graph embedding is a general approach that is used to reduce the computational and storage complexity of graph analytics, and to facilitate processing of graphs by well-known machine learning methods. Graph embedding involves the conversion of graph data into low-dimensional spaces, and allows graphs to be represented in compact vector form (input format preserving relevant network properties) (Cai et al., 2018). Since vector format is one of the most widely used input formats in machine learning algorithms, graph embedding enables downstream analysis with a wide variety of machine learning methods, including methods for clustering and classification. Most graph embedding algorithms are unsupervised learning algorithms with no requirement for labels (or annotations) in the associated graph datasets. This eliminates the time consuming and error prone process of labeling graphs. These characteristics of graph embedding (compact vector representation and unsupervised learning) provide opportunities for developing new applications, such as neural decoding (Chen and Lin, 2018; Lee et al., 2019).

In this work, we focus on graph embedding for graphical models of functional microcircuits (Feldt et al., 2011), which are studied in neuroscience and biomedical engineering. A set of neurons forms a functional microcircuit if (a) they are spatially close to one other (locality condition), and (b) their neural activities are synchronized (Ko et al., 2013) (synchrony condition). Various experimental and computational research works have reported on interactions among neural dynamics of neighboring neurons during cognition and expression of emotion (Fujisawa et al., 2008; Ko et al., 2013; Barbera et al., 2016). Many mechanisms can contribute to the observed synchrony in functional microcircuits. For example, functional microcircuits may reflect synaptically coupled subnetworks. Ko et al. studied somatic calcium signals of nearby layer 2/3 pyramidal neurons in mouse visual cortex *in vivo*, and synaptic connectivity of these neurons *in vitro*. They found that bidirectional synaptic connections were more frequent between neuronal pairs with strongly correlated visual response (Ko et al., 2011). A functional microcircuit can be examined by using calcium imaging (Chen and Lin, 2018).

For computational analysis, functional microcircuits are represented by graphs, which we refer to as *microcircuit models*. Microcircuit models can be in the form of weighted or unweighted graphs. Vertices in these models correspond to neurons, and edges connect pairs of neurons that satisfy or are estimated to satisfy the locality and synchrony conditions described above. The graphs may be directed or undirected, and can consist of several hundreds of vertices. In a widely used framework to derive microcircuit models, the connectivity (edge weight) between a pair of neurons is quantified by a correlation coefficient (with respect to some functional behavior) (Averbeck et al., 2006). Vertices in a functional microcircuit represent the biological cells, the so-called neurons. The vertices are, therefore, generally not interchangeable. In the context of graph embedding, this means that the identities of the vertices must be taken into account in the embedding process.

In this work, we develop a novel graph embedding method for microcircuit models that is based on deep learning (DL). DL has achieved excellent performance levels in a great variety of research fields, such as computer vision and natural language processing. This work represents the first work, to our knowledge, in applying and optimizing DL for the problem of graph embedding for microcircuit models.

Two general classes of graph embedding methods are vertex-level embedding methods, which generate a separate feature vector for each vertex, and graph-level embedding methods, which generate a single feature vector for the whole graph. In this work, we are interested only in graph-level embedding methods due to their importance in analyzing microcircuit models, where the primary interest is on understanding the microcircuit as a whole rather than on finer grained understanding of individual neurons. In the remainder of this paper, when we refer to “graph embedding,” we specifically mean “graph-level embedding” unless otherwise specified.

Representative methods in the literature for graph embedding include DeepWalk (Perozzi et al., 2014), node2vec (Grover and Leskovec, 2016), graph2vec (Narayanan et al., 2017), and PowerGNN (Xu et al., 2018). Among these, graph2vec is especially effective. graph2vec is an unsupervised learning method that employs random walks together with Doc2Vec (Le and Mikolov, 2014), which is a popular DL-based method for natural language processing. In graph2vec, a feature extractor is used to generate a corpus of graphs, and then Doc2Vec converts this corpus into a vector representation. On various benchmark datasets, graph2vec has been shown to provide better performance compared to alternative methods, including node2vec (Grover and Leskovec, 2016), sub2vec (Adhikari et al., 2018), Weisfeiler-Lehman graph kernels (Shervashidze et al., 2011), and deep Weisfeiler-Lehman graph kernels (Yanardag and Vishwanathan, 2015).

Existing graph embedding methods are not well-suited to generating graph embeddings for microcircuit models. First, many graph embedding methods focus on unweighted graphs (Narayanan et al., 2017; Xu et al., 2018; Gutiérrez-Gómez and Delvenne, 2019), whereas, as mentioned previously, both unweighted and weighted graphs are generally relevant in the analysis of microcircuit models. The inability to handle weighted graphs therefore makes a graph embedding approach too restrictive for the objectives of our work, which is to develop a general framework that provides efficient graph embedding for microcircuit models. Similarly, many graph embedding methods, including the popular graph2vec and PowerGNN methods, do not take the identities of vertices into account. To the best of our knowledge, the graph embedding approach presented in this paper is the first to simultaneously support weighted graphs, and take into account the identities of vertices. This makes it uniquely well-suited for application to microcircuit models.

To address the limitations of existing graph embedding algorithms and leverage the capabilities of DL, we aim to develop a new DL-based, graph-level embedding algorithm that is effective for microcircuit models. To this end, we develop in this paper a new unsupervised learning algorithm called Weighted Graph Embedding with Vertex Identity Awareness (WGEVIA). WGEVIA can generate embeddings for both weighted and unweighted graphs, and can also account for vertex identities. Through extensive experiments, we show that the graph embeddings generated by WGEVIA are more effective than previously developed graph embedding methods for analysis of microcircuit models—specifically, for the problem of behavior classification from calcium-imaging-based microcircuit data.

## 2. Methods

In this work, we are interested specifically in an important class of microcircuit models called *coherence-based* models (Zohary et al., 1994; Averbeck et al., 2006). In this context, coherence is a directionless association between pairwise neurons. The coherence association models the synchrony between neuron pairs with respect to the enclosing functional microcircuit. The degree of coherence or synchrony between a pair of neurons can be quantified by a metric, such as the correlation, partial correlation, mutual information or maximal information coefficient. In the remainder of this paper, when we use the term “microcircuit model,” we mean a coherence-based model, unless otherwise stated.

Although this work is focused on coherence based microcircuit models, we envision that it provides a valuable foundation that can be extended to other types of microcircuit models, including those that involve directional associations between neurons. Such extensions represent an interesting direction for future work.

### 2.1. Objective

As described in section 1, a microcircuit model is a graphical model of a functional microcircuit. Coherence-based models use a form of graph called an *undirected graph*. An undirected graph is an ordered pair *G* = (*V, E*), where *V* is a finite set whose elements are referred to as *vertices*, and each element of *E* is a pair {*x, y*} of vertices (*x, y* ∈ *V*). Each element of *E* is called an *edge*. In the remainder of this paper, when we use the term “graph,” we mean an undirected graph, unless otherwise stated.

A *microcircuit model* is an ordered pair *M* = (*G, W*), where *G* is a graph, and *W*:*E* → ℝ is a function that maps the set *E* of edges (in *G*) into the set ℝ of real numbers. We refer to *G* and *W* as the *graph and weight function associated with* *M*. The vertex set and edge set of *G* are denoted as *vertices*(*M*) and *edges*(*M*), respectively. Also, given an edge *e* = {*x, y*}, we say that the vertices *x* and *y* are *incident* to *e*, and we say that *x* and *y* are *neighbors* (*x* is a neighbor of *y* and vice versa). The number of edges that are incident to a given vertex *x* is called the *degree* of the vertex, and is denoted as *deg*(*x*).

In our experiments, the weight of a microcircuit edge is taken to be the Spearman correlation coefficient (McDonald, 2014) between the neurons that are incident to the edge. However, the proposed methods are not specific to use of the Spearman correlation coefficient, and alternatively, other methods for assigning edge weights can be used, such as the partial correlation, mutual information or maximal information coefficient.

We say that a *weighted graph* is a graph with a real-valued quantity called the *weight* (or *edge weight*) associated with each edge in the graph. Thus, a microcircuit model (*G, W*) can be viewed as a weighted graph with the weight of each edge *e* defined to be *W*(*e*).

A *microcircuit dataset* *D* is a set *D* = {*M*[1], *M*[2], …, *M*[*D*_*n*_*G*__]}, where *M*[1], *M*[2], …, *M*[*D*_*n*_*G*__] are microcircuit models with a common vertex set. We denote the common vertex set of *D* as *Vertices*(*D*); thus, *vertices*(*M*[*i*]) = *Vertices*(*D*) for all *i*.

The core contribution of this work is a novel algorithm, called WGEVIA (Weighted Graph Embedding with Vertex Identity Awareness), that takes as input a microcircuit dataset *D* and a positive integer *m*, and outputs a mapping θ*m* :*D* → ℝ^*m*^. The mapping is derived in such a way so that machine learning tasks (*downstream analysis tasks*) can operate efficiently and accurately using the *embedded dataset* {θ*m*(*d*) ∣ *d* ∈ *D*}. A mapping of this form (from a microcircuit *D* into ℝ^*m*^ for some *m*) is called a *microcircuit embedding*. More generally, it can also be viewed as an embedding of a set of weighted graphs; however, the motivation and experiments in this paper are developed in the specific context of microcircuit models.

WGEVIA applies a novel algorithm, called UGEVIA (Unweighted Graph Embedding with Vertex Identity Awareness), which we develop to compute embeddings of unweighted graphs. An embedding θ*u* for unweighted graphs, which we refer to as a *graph embedding*, maps a set of graphs *U* = {*G*[1], *G*[2], …, *G*[*n*_*G*_]} into ℝ^*m*^ for some positive integer *m*—θ*u*:*U* → ℝ^*m*^.

The remainder of this section is summarized as follows. We first discuss the problem of retaining vertex identities when performing graph embedding, and we introduce a novel dataset called the five vertices problem dataset. Next, in sections 2.3–2.5, we introduce in detail the overall UGEVIA algorithm, the feature extraction algorithm used in the UGEVIA algorithm, and the WGEVIA algorithm, respectively. Then in section 2.6, we summarize our approach for evaluating the effectiveness of the proposed new embedding methods for microcircuit models.

### 2.2. Identities of Graph Vertices

An important characteristic of microcircuit models is that each vertex represents a unique neuron, and in general, the identities of the neurons and not just the connectivity patterns among them are relevant to microcircuit analysis methods.

The *five vertices problem* is a simplified benchmark problem that we have designed to help study the interaction between graph embedding and vertex identity awareness. In our experiments, we use this benchmark to assess how effective a graph embedding approach is in terms of taking vertex identities into account.

**Figure 1:**
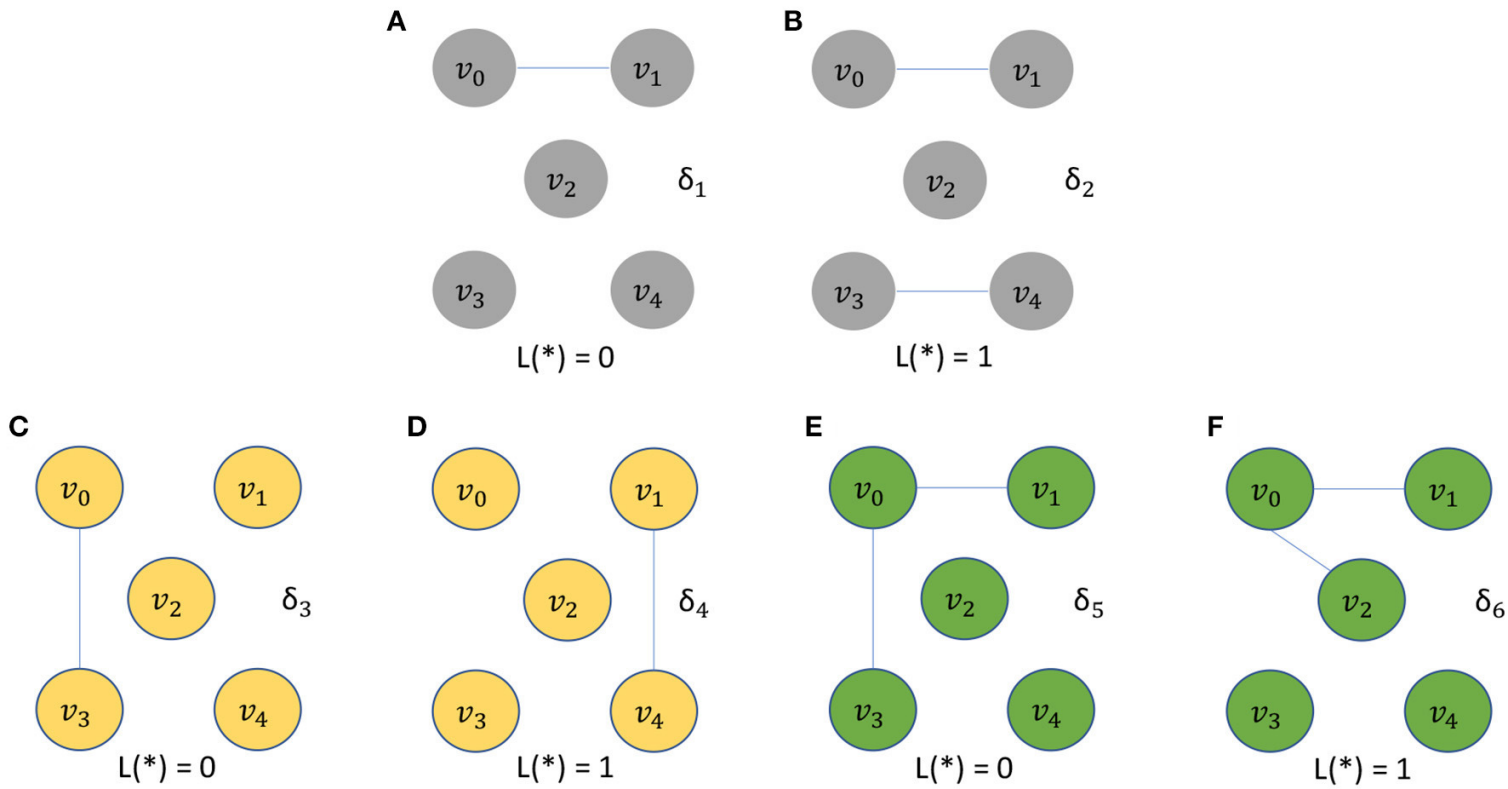
The five vertices problem is a binary classification problem involving a dataset Δ of 3,000 unweighted graphs. All graphs in Δ have a common vertex set *V*_Δ_ = {*v*_0_, *v*_1_, *v*_2_, *v*_3_, *v*_4_}. The structures of graphs with label 0 are depicted in **(A,C,E)**. The structures of graphs with label 1 are depicted in **(B,D,F)**.

The five vertices problem is a binary classification problem involving a dataset Δ of 3,000 unweighted graphs. The dataset, called the *five vertices dataset*, is a union of six subsets Δ = δ_1_ ∪ δ_2_ ∪ … ∪ δ_6_, where each subset δ_*i*_ consists of 500 identical unweighted graphs. We incorporate a large number of graphs, even though they are identical, because the downstream classifier needs a sufficiently large number of instances for proper training. At the same time, in the design of this dataset, we would like to make the two graph classes for each classification problem as simple as possible—this helps to isolate the ability of a given algorithm to take into account vertex identities (without introducing other complicating factors to the classification problem). All 3,000 unweighted graphs in Δ have a common vertex set *V*_Δ_ = {*v*_0_, *v*_1_, *v*_2_, *v*_3_, *v*_4_}. The structures of the graphs in δ_1_, δ_2_, …, δ_6_ are illustrated in Figure 1. Each graph *g* ∈ Δ is assigned a binary label *L*(*g*), where *L*(*g*) = 0 for *g* ∈ (δ_1_ ∪ δ_3_ ∪ δ_5_), and *L*(*g*) = 1 for *g* ∈ (δ_2_ ∪ δ_4_ ∪ δ_6_).

Now consider a graph embedding generator γ′, which operates on elements of the dataset Δ, and provides input to a classifier *C*, where *C* is designed to learn *L* based on some partitioning of Δ into training and testing subsets *d*_*tr*_ and *d*_*te*_, respectively.

Intuitively, it is easy for *C* to distinguish between graphs in δ_1_ and δ_2_ even if γ′ does not take vertex identities into account. On the other hand, without the use of vertex-identity awareness in γ′, it is impossible for *C* to distinguish between graphs in δ_3_ and δ_4_ and between graphs in δ_5_ and δ_6_. This is because of the isomorphic relationship between graphs in the subset pairs δ_3_/δ_4_ and δ_5_/δ_6_.

Now consider the accuracy of the (γ′, *C*) combination in classifying graphs in *d*_*te*_ that belong to $\delta^{\prime }=\left(\delta_{3}\cup \delta_{4}\cup \delta_{5}\cup \delta_{6}\right)$. This accuracy measurement can be considered as an assessment of the effectiveness of γ′ in taking vertex identities into account in the embedding process. In particular, if γ_1_ and γ_2_ are alternative graph embedding generators, then comparing the accuracy of (γ_1_, *C*) and (γ_2_, *C*) on δ′ provides a useful assessment of the relative effectiveness between γ_1_ and γ_2_ in taking vertex identities into account.

At the same time, the subsets δ_1_ and δ_2_ help to provide a comparison between γ_1_ and γ_2_ for scenarios in which vertex identity information is not critical.

In summary, the five vertex dataset is a novel dataset for assessing graph embedding algorithms. The primary emphasis in this dataset is to help assess the effectiveness of an embedding technique in taking vertex identity information into account. The dataset is of special utility in computational neuroscience because neurons that are represented in microcircuit models often need to be distinguished in analysis that is performed on these models.

### 2.3. UGEVIA: Identity-Aware Embedding for Unweighted Graphs

The proposed UGEVIA algorithm can be viewed as a modification to the algorithm used by graph2vec. Our developed modification is aimed at incorporating vertex-identity awareness, and further improving embedding quality.

The graph2vec (Narayanan et al., 2017) framework for graph embedding is designed for unweighted graphs. The framework is based on Weisfeiler-Lehman Graph Kernels (Shervashidze et al., 2011), and it also applies Doc2Vec (Le and Mikolov, 2014), as described in section 1. In graph2vec, a feature extractor based on Weisfeiler-Lehman Graph Kernels is used to generate a collection of text strings (strings) from the given input graphs to be embedded. Such a collection of strings is referred to as a *corpus*. Doc2Vec is then applied to convert the generated corpus into vector representations for the input graphs.

UGEVIA takes as input an indexed set $G^{u}=\left\{g_{1}^{u},g_{2}^{u},\ldots ,g_{n_{G}}^{u}\right\}$ of unweighted graphs which share a common indexed vertex set, and a positive integer *m*, which gives the dimension for the output embedding that is to be computed. The common indexed vertex set for the input graphs is denoted as, employing a minor abuse of notation, *Vertices*(*Gu*) = {*v*_1_, *v*_2_, …, *v*_*k*_}, and the index of a given vertex *v*_*i*_ is referred to as its identifier (ID) and denoted by *ID*(*v*_*i*_) [i.e., *ID*(*v*_*i*_) = *i*]. UGEVIA also takes as input a positive integer *featureGenIters*, which specifies the number of iterations with which to execute the feature extractor on each input graph; this input is discussed further in section 2.4.

UGEVIA produces as output an indexed set $\Omega^{u}=\left\{\nu_{1}^{u},\nu_{2}^{u},\ldots ,\nu_{n_{G}}^{u}\right\}$ of vectors in ℝ^*m*^, where each $\nu_{i}^{u}\in {\mathbb{R}}^{m}$ is the constructed embedding for $g_{i}^{u}$.

As in graph2vec, all of the vertices in all of the input graphs are initially labeled with the associated vertex degrees. The major differences between UGEVIA and graph2vec, which are designed to make the approach more suitable for microcircuit models, are summarized as follows.

- The input to UGEVIA includes unique label IDs, and the feature extraction process utilizes these IDs so that they are taken into account in the generated embeddings.
- Non-zero-degree vertices are labeled with the index of the vertex, while zero-degree vertices are labeled with the character “*Z*” along with the index of the vertex.
- Within each iteration of feature extraction, features of non-zero-degree vertices are updated with features of their connected neighbors. Unique features of the input graph will be generated based on the relative indexing of zero-degree vertices.

The special treatment of zero-degree vertices in UGEVIA preserves the identities of zero-degree vertex subsets, and avoids problems with random-walk approaches when zero-degree vertices are encountered—in particular, random-walk based approaches typically get stuck at zero-degree vertices (if the walks start at such a vertex) or can never reach such a vertex (if they start at a vertex with positive degree). UGEVIA's careful handling of zero-degree vertices is important in computational neuroscience applications because microcircuit models are often sparse, and therefore contain a large proportion of zero-degree vertices. Although many vertices have zero degree, the associated neuron identities must be taken into account for accurate analysis of microcircuit models.

Algorithm 1 presents a pseudocode representation of the UGEVIA algorithm. Here, *featureDoc* is a list of ordered pairs having the form (*g*, β), where *g* is a graph, and β is a list of strings that provide a feature representation for *g*. A feature set, represented as a text string, is associated with each vertex in each $g_{i}^{u}$.

**Algorithm 1:**
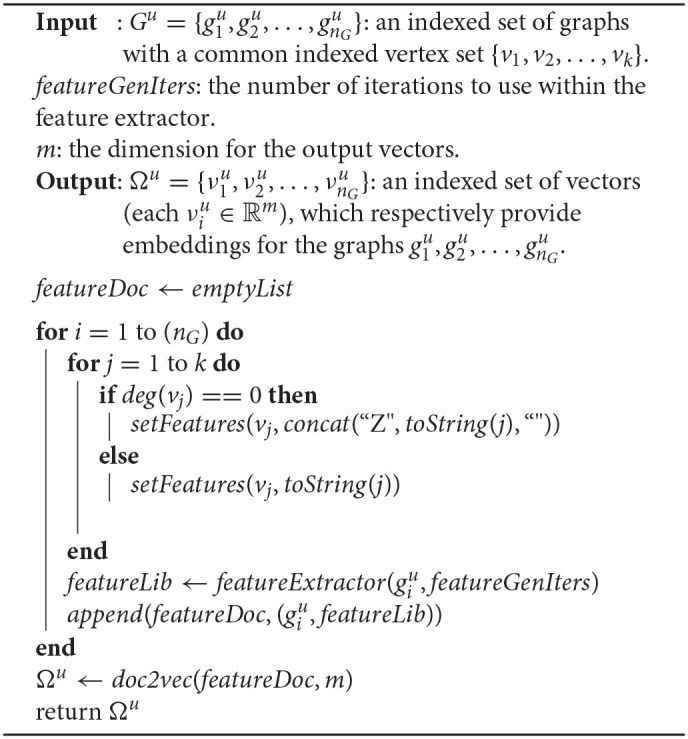
A pseuodcode representation of the UGEVIA algorithm.

The function *setFeatures*(*v, s*) sets the features of vertex *v* to be the string *s*. The feature set associated with a vertex, as set by function *setFeatures*, can be retrieved or overwritten by the UGEVIA feature extractor (see section 2.4) using the *getFeatures* or *setFeatures* functions, respectively.

The function *concat*(*s*_1_, *s*_2_, *delim*) returns the concatenation of string *s*_1_ followed by string *delim* (called the delimiter string), and then followed by string *s*_2_. For example, *concat*(“27”, “33”, “_”) returns “27_33”, which is a delimited concatenation of the string representations for two integers. The function *toString*(*x*) returns the string representation of the integer *x*.

The function *featureExtractor*(*g, I*) returns a list of strings as features for the graph *g*. The feature extraction process is parameterized with a number of iterations; the argument *I* provides the setting for the iteration count parameter. More details on the algorithm underlying function *featureExtractor* are discussed in section 2.4.

The function *append*(κ, *elem*) in Algorithm 1 adds the new list element *elem* (an ordered pair in this particular use of the function) to the end of the list κ.

The function *doc2vec* in Algorithm 1 calls the off-the-shelf Doc2Vec utility, which has been described above. The *doc2vec* wrapper utilizes certain configuration parameters for Doc2Vec; settings for these parameters in our experiments are discussed in section 3.

### 2.4. UGEVIA Feature Extractor

The feature extractor for UGEVIA takes as input a graph *g*^*u*^ with an ordered vertex set {*v*_1_, *v*_2_, …, *v*_*k*_}. It is assumed that each vertex has an initial feature set, which is accessible using the *getFeatures* function (see section 2.3). The algorithm also takes as input a positive integer *featureGenIters*, which as mentioned previously, controls the number of iterations to employ in the feature extraction process. The feature extractor produces as output a list of strings *featureLib*, called a *feature library*, that provides a feature set for representation of the input graph *g*^*u*^.

Use of a larger value of *featureGenIters* results in longer computation time and more storage required for feature extraction, but it may also lead to higher quality feature sets, which improve the accuracy of downstream classification tasks. In our current approach to applying UGEVIA, we determine the value of *featureGenIters* empirically so as to optimize accuracy up to a point of diminishing returns—where additional iterations increase computational time with negligible accuracy improvement. In our experiments, we used the value *featureGenIters* = 4, which is found by Bayesian optimization. With the value *featureGenIters* = 4, the extracted features are effective for all of the datasets involved in our experiments.

During each feature extraction iteration, the feature extractor updates features for each vertex *v*_*i*_, and appends new features to the feature library. If a vertex has non-zero degree, its features are updated using the features of its neighbors in a given feature extraction iteration. On the other hand, if a vertex has zero degree, its features are updated with the features of the vertices that immediately precede and succeed it within the ordering *v*_1_, *v*_2_, …, *v*_*k*_. This is an additional way in which the identities of vertices are taken into account in the graph embedding process—different pairs of preceding and succeeding vertices have distinct pairs of vertex IDs. The determination of preceding and succeeding vertices is performed in a wrap-around sense—i.e., using the interpretation that *v*_*k*_ immediately precedes *v*_1_, and equivalently, that *v*_1_ immediately succeeds *v*_*k*_.

The method described above is tolerant to rearrangement of the vertex sequence in the common vertex set. The algorithm is tolerant to such rearrangement because the algorithm requires only that each vertex has a unique label, and that the vertex labels are consistent across all graphs. The consistency of the labels allows the algorithm to extract features associated with graph structure.

A pseudocode representation of the UGEVIA feature extractor, encapsulated by the function *featureExtractor*, is shown in Algorithm 2. Some functions, such as *concat* and *getFeatures*, have been discussed already in section 2.3; in the remainder of this section, we define the functions used in Algorithm 2 that have not already been discussed.

**Algorithm 2:**
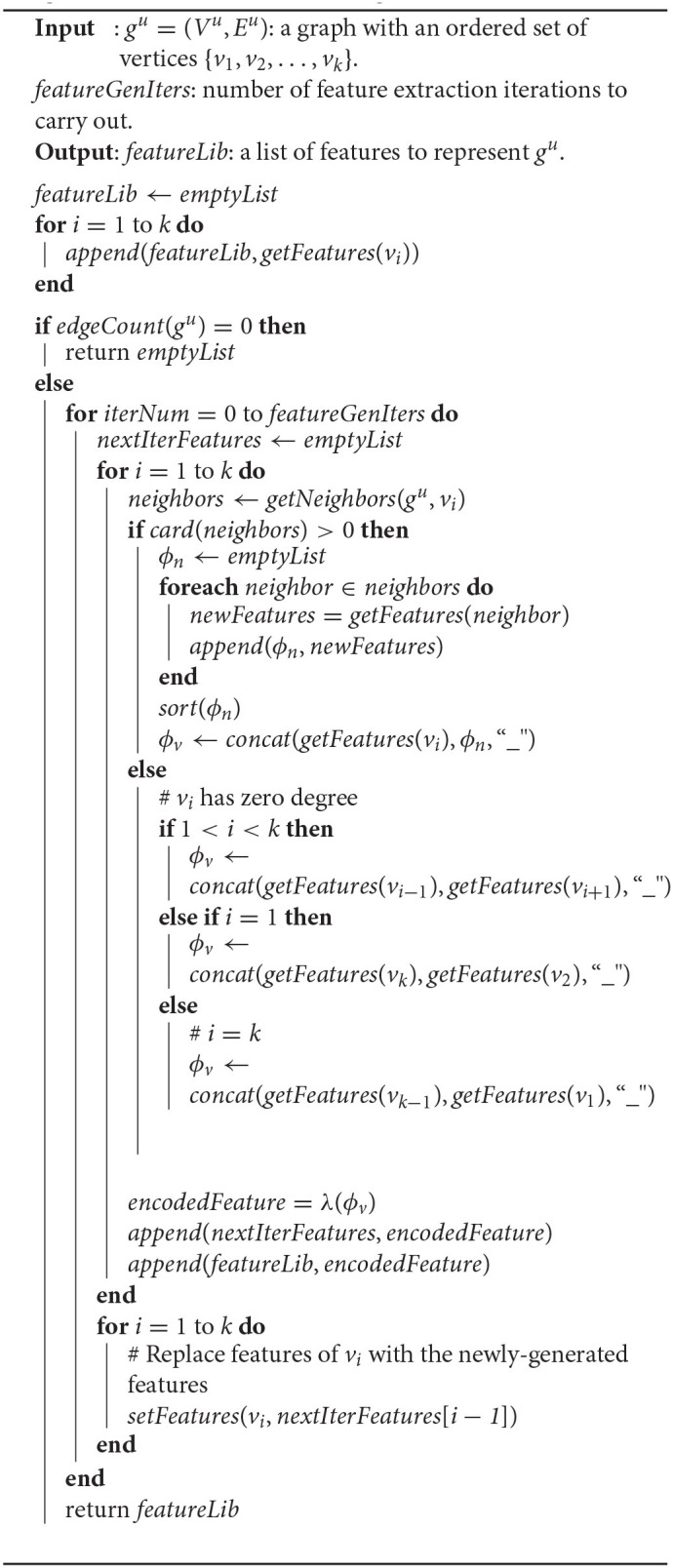
Feature extraction algorithm for UGEVIA.

The function *edgeCount*(*g*) returns the number of edges in the graph *g*. The function *getNeighbors*(*g, v*) returns the set of neighbors of a given vertex *v* in a given graph *g*. The function *card*(*S*) returns the cardinality of the set *S*. The function *sort*(*list*) modifies the list of strings *list* by sorting its elements.

The function λ(*s*) first employs a hash function to convert a string *s* of arbitrary length into a 128-bit representation. The function then converts the 128-bit representation into a compact, fixed-length string representation of 32 characters. The hash function used is the MD5 hash function (Rivest, 1992). The 32-character string representation is derived as a string representation of the hexadecimal number for the 128-bit output of the hash function. The function λ is used to avoid excessive storage size for vertex and graph features.

### 2.5. WGEVIA: A Multi-Channel Approach for Microcircuit Datasets

UGEVIA is not suitable for microcircuit datasets because although it takes vertex identities into account, the algorithm does not take into account graph weights. To simultaneously take into account vertex identities and graph weights, we develop the WGEVIA algorithm.

A simplified description of the inputs to WGEVIA was given in section 2.1. The full input list along with an output specification, and a pseudocode representation is shown in Algorithm 3. Since a microcircuit dataset is defined as an indexed set of weighted graphs, WGEVIA can be applied to many other application areas that employ weighted graphs; the underlying method is not limited to applications in neural signal processing and computational neuroscience.

**Algorithm 3:**
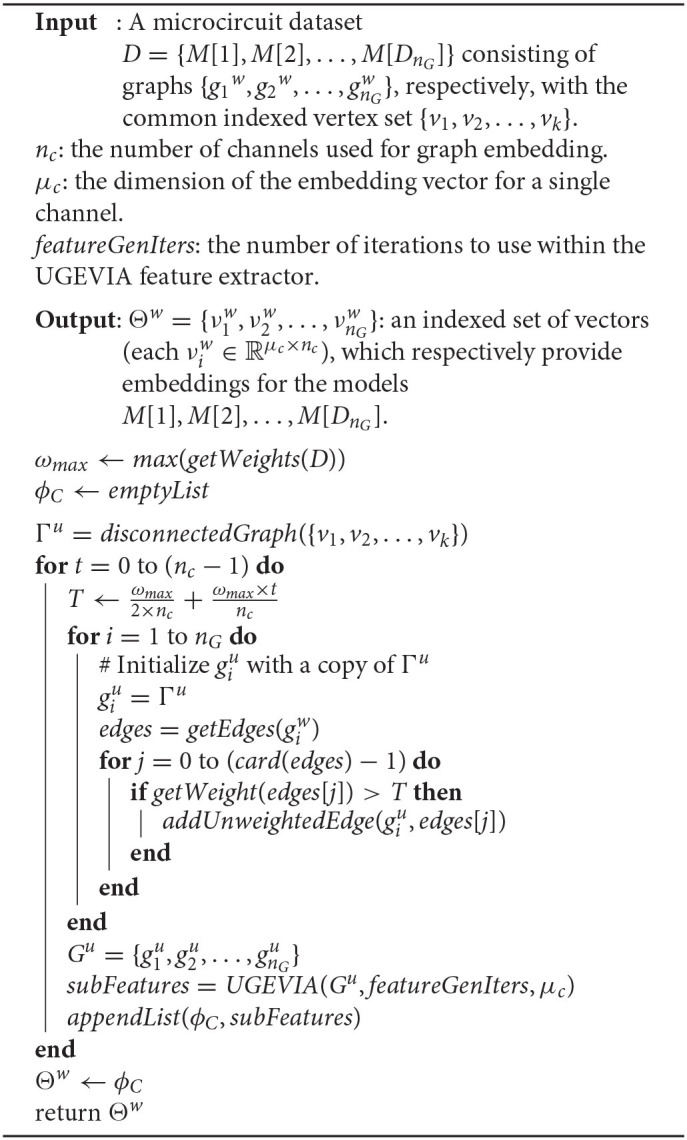
A pseuodcode representation of the WGEVIA algorithm.

WGEVIA repeatedly applies UGEVIA on sets of unweighted graphs, called *channels*, that are derived from the input microcircuit dataset. The use of channels in WGEVIA is inspired by the common use of channelization in convolutional neural networks (e.g., channelization of networks to process red, blue and green components of color images). Intuitively, channels in WGEVIA are derived by applying a threshold to the edge weights and retaining only those edges in a channel whose weights exceed the threshold. As shown in Algorithm 3, the threshold starts from $\frac{w_{max}}{2\times n_{c}}$, increments by $\frac{w_{max}}{n_{c}}$, and stops at $\frac{w_{max}}{2\times n_{c}}+w_{max}\times \frac{n_{c}-1}{n_{c}}$, where *n*_*c*_ is the number of channels. For example, if the edge weights are uniformly distributed in [0, 1], and the number of channels is 10, then according to method of how to set the threshold in Algorithm 3, the first channel will have 95% of edges remaining, the second channel will have 85% of edges remaining, and the tenth channel will have 5% of edges remaining.

Note that the dimension of the output embedding vectors for WGEVIA is not specified explicitly; instead, it is derived as (μ*c* × *nc*), where *nc* is the number of channels to be employed in WGEVIA, and μ*c* is the embedding vector dimension used on each channel when invoking UGEVIA.

We tune the WGEVIA parameters μ*c* and *nc* experimentally. Through empirical analysis together with Bayesian optimization (Pelikan et al., 1999), we derived the values *nc* = 10 and μ*c* = 8 for use in our experiments.

In Algorithm 3, the function *max*(*S*) returns the maximum value from a given set *S* of real numbers. The function *getWeights*(*H*) returns the set of all of the unique edge weight values (i.e., a set of real numbers) across all of the weighted graphs within the given microcircuit dataset *H*. The function *disconnectedGraph*(*V*) takes as argument a set *V* of vertices and returns an unweighted graph whose vertex set is *V* and whose edge set is empty. The function *getEdges*(*g*) returns the set of edges in the weighted or unweighted graph *g*. The function *getWeight*(*e*) returns the weight of the given edge *e*. The function *addUnweightedEdge*(*g, e*) takes as argument an unweighted graph *g* and a weighted edge *e*. The function modifies *g* by adding an unweighted version of *e* to the edge set of *g*. An unweighted version of *e* is simply an edge that is incident to the same pair of vertices, but has no associated weight. The function *appendList*(*L*_1_, *L*_2_) modifies the list *L*_1_ by appending to it all of the elements in *L*_2_. The resulting *L*_1_ is the concatenation of the original *L*_1_ with *L*_2_.

The approach in WGEVIA for deriving thresholds can be modified in various ways. For example, instead of setting the minimum threshold to be $\frac{\omega max}{2\times nc}$, one can generate thresholds within the range (ω_*min*_, ω_*max*_), where ω_*min*_ is the minimum weight across all edges in the microcircuit dataset. Similarly, instead of using uniformly-spaced thresholds, one can distribute the thresholds non-uniformly using more sophisticated computations to determine the inter-threshold spacings. Investigation into strategies for adapting the threshold derivation in WGEVIA is beyond the scope of the paper. This is another interesting direction for future work.

### 2.6. Graph Classification

We demonstrate the effectiveness of WGEVIA by applying it to two different microcircuit classification problems and studying its performance when it is connected to four different classifiers for each classification problem.

Both of the classification problems that we study are binary classification problems, where the objective involves discriminating between 0- and 1-valued labels for each microcircuit model. The first classification problem, which is based on data generated from a real-world calcium imaging study, involves a dataset that we refer to as the REAL dataset. The second classification problem involves a dataset generated using simulation techniques; we refer to it as the SIMU dataset. Section 3.4 provides more details on these microcircuit classification problems, and the associated datasets.

The four different types of classifiers used in our experiments are:

- single-mlp: a multilayer perceptron (MLP) (Gardner and Dorling, 1998) with one hidden layer.
- multi-mlp: an MLP with two hidden layers.
- SVM-rbf: a support vector machine (SVM) (Boser et al., 1992) with Radial Basis Function (RBF) kernel.
- LDA: a Linear Discriminant Analysis (LDA) classifier (Balakrishnama et al., 1998).

The classifiers are used in the experiments as downstream analysis tasks that operate on the graph embeddings generated by WGEVIA. The experiments with these classifiers demonstrate the improvements in classification accuracy enabled by WGEVIA when applied to our two different microcircuit classification problems. The use of two different classification problems and a variety of classifiers in the experiments helps to demonstrate the general utility of WGEVIA in enhancing the performance of different types of downstream analysis subsystems.

## 3. Experimental Results

In this section, we present experiments that demonstrate the effectiveness of our proposed graph embedding framework for microcircuit models. The section is organized as follows. First, section 3.1 describes two baseline methods that we use for comparison purposes in our experiments. Next, section 3.2 describes four different classifiers that we use as downstream analysis tasks to demonstrate the application of WGEVIA to a variety of different analysis subsystems. Then section 3.3 describes common settings of the doc2vec utility that we employ in our experiments (doc2vec is used within graph2vec and WGEVIA). Section 3.4 describes microcircuit datasets used in our experiments. Section 3.5 evaluates graph2vec, PowerGNN and UGEVIA on the five vertices problem described in section 2.2. Recall from section 1 that graph2vec and PowerGNN are two state-of-the-art methods for graph embedding. In section 3.6, we present a quantitative evaluation comparing WGEVIA with graph2vec and PowerGNN. In section 3.7, comparison experiments are presented to assess the impact of selected design decisions in section 2. Section 3.8 shows how visualization can be used to gain intuitive insight into the results produced by WGEVIA. Section 3.9 examines the runtime of the proposed algorithms and studies hyperparameter tuning. Our algorithms are implemented with Python 3.

### 3.1. Baseline Methods

We apply graph2vec and PowerGNN, which were introduced in section 1, as two baseline graph embedding methods to compare with the proposed UGEVIA and WGEVIA methods. Both graph2vec and PowerGNN are state-of-the-art graph embedding methods for unweighted graphs. Recall that graph2vec is also a foundation on which the UGEVIA method builds. For our comparisons with graph2vec, we use the popular graph2vec implementation by Rozemberczki et al. (2020).

PowerGNN is a specialized graph embedding method for graph classification applications. Unlike graph2vec, UGEVIA, and WGEVIA, a classifier is integrated within PowerGNN. The fully connected layers in a PowerGNN model can be considered as its classifier. The fully connected layers are similar to a multi-layer perceptron (MLP) introduced in section 3.2. Although PowerGNN uses a graph embedding technique internally, it outputs only the classification results, not the intermediate embedding results. Thus, in our experiments, we apply PowerGNN as a combined embedding + classification method without adding any downstream classifier to it. We use a Bayesian optimization technique to tune the hyperparameters of PowerGNN.

### 3.2. Downstream Classifiers

All reported accuracy scores are from classification tasks on input graphs with embedding vectors generated by different graph embedding methods. The embedding vectors are the input to classification tasks. The embedding vector is a representation of the whole graph. Each element of the vector is a floating point value. Graph embedding quality is assessed in terms of the accuracy of classifiers that operate on the generated embeddings. To ensure that our measurement of graph embedding quality is not specific to a particular type of downstream classifier, we used four different downstream classifier models: single-mlp, multi-mlp, SVM-rbf, and LDA, as described in section 2.6.

The single-mlp model has one hidden layer with 128 nodes with a RELU activation function. The output layer has two nodes with a Softmax activation function. The multi-mlp model has two hidden layers. The first hidden layer has 128 nodes with a RELU activation function. The second hidden layer has 64 nodes, also with a RELU activation function. The output layer is the same as that of the single-mlp classifier.

The Adam optimizer is used to train the single-mlp and multi-mlp models (Kingma and Ba, 2014). Both models are constructed with TensorFlow (Abadi et al., 2016), and trained for 100 epochs each. The SVM-rbf classifier has two hyperparameters, *C* and γ; we refer readers to Han et al. (2012) for more details about these two hyperparameters in this classifier. We have tuned the SVM-rbf model using grid search to determine the optimized hyperparameter values *C* = 100 and γ = 0.1. For the LDA classifier, we used the same grid search strategy to tune the tolerance *tol*; the value we derived for this hyperparameter is *tol* = 0.0001. For each of the four classifiers, we fixed the structure and hyperparameters across all experiments to help derive fair comparisons across different graph embedding methods.

All models are trained with a Tesla K40c GPU with 12 GB memory and an Intel Xeon E5-2620 v4 CPU with 128 GB memory. All reported accuracy results are based on 10-fold cross-validation (Refaeilzadeh et al., 2009). For each round of cross validation, the input dataset is randomly divided into training and test sets. The random division is performed in such a way that there is no overlap between the test sets of different rounds.

### 3.3. Doc2Vec Settings

As described in section 2, the Doc2Vec algorithm is used both in graph2vec and WGEVIA. Doc2Vec involves many hyperparameters. We carefully tuned the hyperparameters and used the same hyperparameters for the Doc2Vec model used inside both graph2vec and WGEVIA. Specifically, we set the learning rate to 0.025, downsampling rate to 0.0001, minimum count to 1, and number of epochs to be 100. The minimum count provides a threshold that Doc2Vec uses to determine which words to ignore. All words with total frequency below this threshold are ignored. The downsampling rate is a threshold that Doc2Vec uses to downsample high-frequency words. We refer the reader to Le and Mikolov (2014) for more details about the hyperparameters of Doc2Vec.

### 3.4. Microcircuit Datasets

We assessed the proposed algorithm on two microcircuit datasets: REAL and SIMU, which were briefly introduced in section 2.6. In this section, we provide more details about these datasets. As mentioned in section 2.6, REAL is generated from a real-world calcium imaging study (Zaremba et al., 2017), while SIMU is generated by simulation. In both datasets, each microcircuit is associated with a binary behavioral variable as its label.

The neuron model used in SIMU is the integrate-and-fire model with additive noise (Gütig and Sompolinsky, 2006). The model can be represented by

(1) $$\begin{array}{r} \frac{d\rho }{dt}=\frac{\rho_{rest}-\rho }{\tau }+\sigma \times \tau \times \left(-0.5\right)\times \varepsilon \end{array}$$

where ρ is the membrane potential, ε is a Gaussian random variable with mean 0 and standard deviation 1, σ is a parameter that controls the noise, τ is the membrane time constant, and ρ_*rest*_ is the resting potential. The membrane potential ρ changes with spikes that are received through the synapses. A neuron generates a spike if ρ is greater than a threshold. The neuron model has refractoriness—i.e., a brief time period is needed between two spikes of a neuron. Such a neuron model can represent post-synaptic potentials described in the literature (Brette et al., 2007).

Our simulation to generate the SIMU dataset included a simulation model consisting of 100 neurons. Neurons in the simulation were divided into two groups: groups A and B. Neurons in group A (50 neurons) had no parent neurons. They were activated by a stimulus. Neurons in group B (50 neurons) were activated by neurons in group A. We simulated two experimental conditions. The simulated data were binary. In condition 1, neurons in group B were activated by one or two neurons in group A. In condition 2, neurons in group B were activated by three or four neurons in group A. Based on the generated ensemble neural activities, we calculated the Spearman correlation coefficient of two neurons' activities within a time window. The Spearman correlation coefficient between a neuron pair is calculated based on the signals of the two neurons in a time window. SIMU has 580 graphs labeled as “0” and 583 microcircuit models labeled as “1,” where these labels correspond to conditions 1 and 2, respectively. Details about the microcircuit generation process employed to generate SIMU are described in Chen and Lin (2018).

The REAL dataset was generated from the reward zone study in Zaremba et al. (2017). This dataset (id: jz121, 2015-02-21-16h06m) was originally used in Zaremba et al. (2017). We reanalyzed this dataset. The experimental procedures conducted in that study involving animals were approved by the Columbia University Institutional Animal Care and Ethics Committee. A mouse licked to receive water rewards when entering a fixed reward zone on a treadmill belt. The context considered involved the environment and a set of features during the experiment, including the cues, fabric belts, and non-spatial odor. The animal was trained to learn the reward zone location during context presentation. A reward zone was a 20-cm region in a 2-m long treadmill belt. Two-photon imaging was used to image the CA1 pyramidal layer. There were 21 trials. Details about the animal, virus, surgical procedure, behavioral training, stimulus presentation, and *in vivo* two-photon imaging were discussed in Zaremba et al. (2017). A microcircuit model in this dataset has 420 vertices. The Spearman correlation coefficient between a neuron pair is calculated based on the signals of the two neurons in a time window of 40 frames. The edges are quantified by the Spearman correlation coefficient. The label of a microcircuit model in REAL is a binary behavioral variable that indicates whether or not the mouse is in the reward zone—labels of 1 and 0 correspond to being and not being in the reward zone, respectively. For behavior assessment, only location (whether or not the mouse is in the reward zone) is used and no other behavior variables, such as the animal's velocity, are used. REAL has 1,836 graphs labeled as “1” and 16,036 graphs labeled as “0.” Because REAL is a highly imbalanced dataset, we report the balanced accuracy for experiments involving REAL. The balanced accuracy in our context is the average recall across both classes (label 1 and label 0).

Microcircuits are generated based on a stream of neuron signals which are generated by the neuron detection algorithm. The neuron detection algorithm analyzes the whole data stream and guarantees that the microcircuit graphs will have a common fixed vertices set.

**Figure 2:**
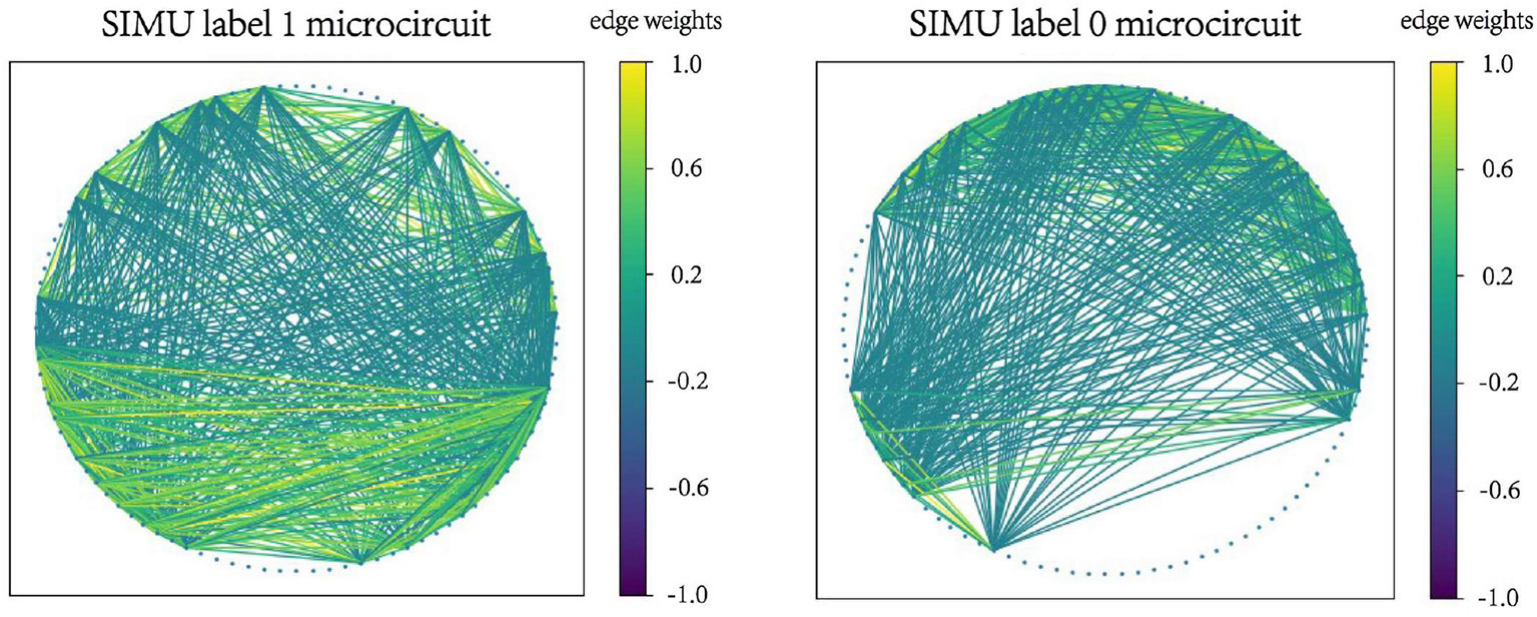
Examples of microcircuits from both label “1” and label “0” classes in the SIMU dataset.

**Figure 3:**
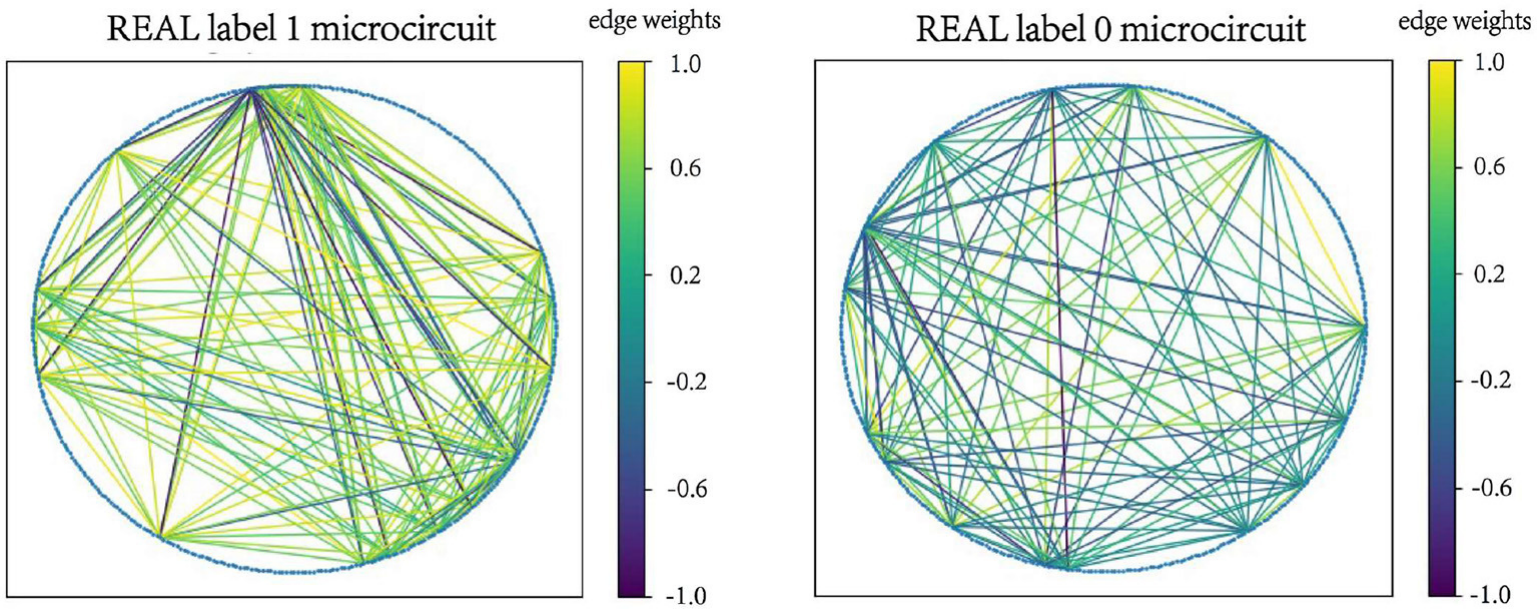
Examples of microcircuits from both label “1” and label “0” classes in the REAL dataset.

Examples of microcircuits from both label “1” and label “0” classes in SIMU and REAL are plotted in Figures 2, 3. The weighted edges are represented as colored connections between neurons with weight values mapped to colors, as shown in the color bars.

### 3.5. Experiments Using the Five Vertices Dataset

We perform experiments using the five vertices dataset (see section 2.2) to evaluate the ability of different graph embedding methods to take vertex identities into account. UGEVIA, graph2vec, and PowerGNN are applied in three comparison experiments involving different subsets of the five vertices dataset Δ. As described in section 2.2, each panel δ_1_, δ_2_, …, δ_6_ in the five vertices dataset Δ contains 500 identical unweighted graphs with structure shown in Figure 1. In the first experiment, δ_1_ and δ_2_ are combined into a set with 1,000 graphs. The first experiment aims to test whether a given graph embedding algorithm can effectively distinguish graphs holding unique vertex identities and different numbers of edges. In the second experiment, δ_3_ and δ_4_ are combined into a set with 1,000 graphs. In the third experiment, δ_5_ and δ_6_ are combined into a set with 1,000 graphs. The goal of the second and third experiments is to test whether a graph embedding algorithm can effectively distinguish graphs with identical numbers of edges but with the edges located between vertices having different identities.

Throughout the remainder of this section, results on classification accuracy are reported with the mean and standard deviation from 10-fold cross-validation. The results are tabulated in the format “*mean*±*standard deviation*.”

The results for the first, second and third experiments are summarized in Tables 1–3, respectively. In Tables 1–3, we use “*” to mark a significant difference between the reference method and the comparison (*p*-value < 0.05, pairwise *t*-test, two-tailed). UGEVIA is the reference method. Note that only one value is reported in each row associated with PowerGNN because PowerGNN uses its own internal classifier instead of the downstream classifier that we use with graph2vec and UGEVIA (see section 3.1). According to the results of the first experiment, graph2vec is not able to fully distinguish between graphs from δ_1_ and δ_2_, whereas UGEVIA and PowerGNN can fully distinguish graphs from these two subsets. From the results of the second and third experiments, we see that graph2vec and PowerGNN are unable to distinguish between graphs from δ_3_ and δ_4_, nor between δ_5_ and δ_6_, respectively. On the other hand, the UGEVIA algorithm can fully distinguish between those pairs of graph subsets. This is because the graphs from δ_3_ and δ_4_ are indistinguishable without a mechanism for vertex identity awareness, and so are the graphs from δ_5_ and δ_6_. The graph2vec and PowerGNN methods lack any such mechanism. For graphs from δ_1_ and δ_2_, although the two groups of graphs contain different numbers of edges, graph2vec treats edges in subsets (a) and (b) similarly, and extracts the same features for all edges. The identical features extracted by graph2vec degrade the final classification performance.

**Table 1:** Results from the first experiment using the five vertices dataset.

	**Test accuracy (Graph set 1)**			
**Algorithm**	**Single-mlp**	**Multi-mlp**	**SVM-rbf**	**LDA**


graph2vec	0.726 ± 0.043*	0.73 ± 0.038*	0.481 ± 0.093*	0.761 ± 0.047*
UGEVIA	1 ± 0	1 ± 0	1 ± 0	1 ± 0
PowerGNN	1 ± 0			
	**Train accuracy (Graph set 1)**			
**Algorithm**	**Single-mlp**	**Multi-mlp**	**SVM-rbf**	**LDA**
graph2vec	1 ± 0	1 ± 0	1 ± 0	0.791 ± 0.008*
UGEVIA	1 ± 0	1 ± 0	1 ± 0	1 ± 0
PowerGNN	1 ± 0			

*Performance results for 10-fold cross-validation are reported in the form mean±standard deviation*.

**Table 2:** Results from the second experiment using the five vertices dataset.

	**Test accuracy (Graph set 2)**			
**Algorithm**	**Single-mlp**	**Multi-mlp**	**SVM-rbf**	**LDA**


graph2vec	0.482 ± 0.057*	0.516 ± 0.063*	0.446 ± 0.037*	0.507 ± 0.052*
UGEVIA	1 ± 0	1 ± 0	1 ± 0	1 ± 0
PowerGNN	0.5 ± 0*			
	**Train accuracy (Graph set 2)**			
**Algorithm**	**Single-mlp**	**Multi-mlp**	**SVM-rbf**	**LDA**
graph2vec	1 ± 0	1 ± 0	1 ± 0	0.625 ± 0.005*
UGEVIA	1 ± 0	1 ± 0	1 ± 0	1 ± 0
PowerGNN	0.5 ± 0*			

**Table 3:** Results from the third experiment using the five vertices dataset.

	**Test accuracy (Graph set 3)**			
**Algorithm**	**Single-mlp**	**Multi-mlp**	**SVM-rbf**	**LDA**


graph2vec	0.489 ± 0.029*	0.514 ± 0.059*	0.453 ± 0.024*	0.51 ± 0.028*
UGEVIA	1 ± 0	1 ± 0	1 ± 0	1 ± 0
PowerGNN	0.5 ± 0*			
	**Train accuracy (Graph set 3)**			
**Algorithm**	**single-mlp**	**multi-mlp**	**SVM-rbf**	**LDA**
graph2vec	1 ± 0	1 ± 0	1 ± 0	0.621 ± 0.011*
UGEVIA	1 ± 0	1 ± 0	1 ± 0	1 ± 0
PowerGNN	0.5 ± 0*			

### 3.6. Graph Classification With Microcircuit Data

We apply WGEVIA, graph2vec and PowerGNN to the SIMU and REAL datasets. Since PowerGNN and graph2vec were designed for unweighted graphs, we introduce a threshold-based method to convert weighted graphs to unweighted graphs before inputting microcircuit models to these two methods. The conversion method uses a hyperparameter χ, which is a threshold for determining whether or not a weighted edge is removed from the graph during the conversion process. In particular, if *W*(*e*) represents the weight of an edge *e* in a microcircuit model, then our weighted-to-unweighted conversion process removes *e* from the graph if *W*(*e*) ≤ χ; otherwise, the edge *e* is retained in the converted graph. In either case, the weight information [*W*(*e*)] is discarded in the conversion process since the objective is to derive an unweighted graph. We tune the hyperparameter χ to maximize accuracy using Bayesian optimization (Pelikan et al., 1999). The hyperparameter value in our experiments that results from this tuning process is χ = 0.196. Hyperparameter settings for WGEVIA that are used in this experiment are: *nc* = 10 and μ*c* = 8.

The results of this experiment using microcircuit data are summarized in Table 4. In Table 4, we again use “*” to mark a significant difference between the reference method and the comparison (*p*-value < 0.05, pairwise *t*-test, two-tailed). Our method WGEVIA is the reference method. From the results, we see that WGEVIA consistently outperforms graph2vec and PowerGNN on both datasets SIMU and REAL. WGEVIA outperforms the other two methods by significant margins for each dataset. This is perhaps not surprising since edge weights are critical to microcircuit analysis, and we expect that we would need to deeply take weight values into account to achieve high accuracy. The results in this experiment help to quantify this intuition, and to validate the effectiveness of WGEVIA in taking edge weights into account.

**Table 4:** Results of WGEVIA, graph2vec, and PowerGNN for graph classification on microcircuit data.

	**Test accuracy (SIMU)**				**Test accuracy (REAL)**			
**Algorithm**	**Single-mlp**	**Multi-mlp**	**SVM-rbf**	**LDA**	**Single-mlp**	**Multi-mlp**	**SVM-rbf**	**LDA**
graph2vec	0.943 ± 0.018*	0.955 ± 0.014*	0.896 ± 0.027*	0.769 ± 0.035*	0.677 ± 0.011*	0.686 ± 0.008*	0.55 ± 0.008*	0.513 ± 0.007*
WGEVIA	1 ± 0	1 ± 0	1 ± 0	0.997 ± 0.004	0.991 ± 0.001	0.993 ± 0.001	0.993 ± 0.001	0.913 ± 0.003
PowerGNN	0.788 ± 0.033*				0.509 ± 0.002*			
	**Train accuracy (SIMU)**				**Train accuracy (REAL)**			
**Algorithm**	**Single-mlp**	**Multi-mlp**	**SVM-rbf**	**LDA**	**Single-mlp**	**Multi-mlp**	**SVM-rbf**	**LDA**
graph2vec	0.999 ± 0	0.999 ± 0	1 ± 0	0.83 ± 0.008*	0.789 ± 0.006*	0.822 ± 0.024*	0.644 ± 0.003*	0.498 ± 0.001*
WGEVIA	1 ± 0	1 ± 0	1 ± 0	0.998 ± 0	0.998 ± 0.001	0.998 ± 0.001	1 ± 0	0.909 ± 0.002
PowerGNN	0.819 ± 0.021*				0.522 ± 0.002*			

### 3.7. Evaluation on Design Decisions

As described in section 2, there are three main improvements incorporated on top of graph2vec in our design of the WGEVIA algorithm: (1) adding vertex identity awareness, (2) designing special features for zero-degree vertices, and (3) utilizing a multi-channel approach, where a weighted graph is processed as multiple unweighted graphs, and each of the unweighted graphs is referred to as a *channel*. In this section, we aim to evaluate the impact of each of these improvements. The algorithms applied in this evaluation are:

- graph2vec, which is the original graph2vec algorithm without any modification except for weighted-to-unweighted graph conversion being applied to its input, as described in section 3.6;
- Multi-Channel graph2vec (MC-graph2vec), which incorporates Modification 3 described above while not incorporating Modifications 1 and 2;
- Multi-Channel Index Labeled graph2vec, which incorporates Modifications 1 and 3, but not Modification 2;
- WGEVIA, which incorporates all three modifications.

Both the SIMU and REAL datasets are used for the evaluation presented in this section. We perform the experiments with two sets of hyperparameters for the multi-channel approaches: (a) *nc* = 10 and μ*c* = 8, and (b) *nc* = 4 and μ*c* = 8. The first set of hyperparameters is the common set that we used for all other experiments involving multi-channel approaches. The second set of hyperparameters makes the generated embedding vectors less informative. The results with the second set help to provide insight into the robustness of the different methods to variations in hyperparameter settings.

The results from the experiments presented in this section are summarized in Tables 5, 6. For both sets of hyperparameters, we see that Modification 3 alone (MC-graph2vec), Modifications 1 + 3, and Modifications 1 + 2 + 3 (WGEVIA) provide progressively better (monotonically increasing) accuracy compared to the original graph2vec approach. Moreover, except for the case of the LDA classifier with the REAL dataset, WGEVIA provides very high accuracy even with the lower-quality hyperparameter configuration in the second hyperparameter set. Even for the LDA classifier and the second hyperparameter set, the results provided by WGEVIA on REAL are significantly better than the original graph2vec method.

**Table 5:** Results from evaluating the impact of modifications incorporated on top of graph2vec in our design of the WGEVIA algorithm—results for *nc* = 10 and μ*c* = 8.

	**Test accuracy (SIMU)**				**Test accuracy (REAL)**			
**Algorithm**	**Single-mlp**	**Multi-mlp**	**SVM-rbf**	**LDA**	**Single-mlp**	**Multi-mlp**	**SVM-rbf**	**LDA**
graph2vec	0.943 ± 0.018*	0.955 ± 0.014	0.896 ± 0.027*	0.769 ± 0.035*	0.677 ± 0.011*	0.686 ± 0.008*	0.55 ± 0.008*	0.513 ± 0.007*
MC-graph2vec	0.973 ± 0.015*	0.97 ± 0.02*	0.938 ± 0.02*	0.851 ± 0.046*	0.753 ± 0.007*	0.794 ± 0.008*	0.726 ± 0.006*	0.569 ± 0.006*
MC-graph2vec (Index Labeled)	0.998 ± 0.003*	0.998 ± 0.003*	0.997 ± 0.008	0.987 ± 0.002*	0.97 ± 0.015*	0.986 ± 0.002*	0.97 ± 0.003*	0.647 ± 0.006*
WGEVIA	1 ± 0	1 ± 0	1 ± 0	0.997 ± 0.004	0.991 ± 0.001	0.993 ± 0.001	0.993 ± 0.001	0.913 ± 0.003
	**Train accuracy (SIMU)**				**Train accuracy (REAL)**			
**Algorithm**	**Single-mlp**	**Multi-mlp**	**SVM-rbf**	**LDA**	**single-mlp**	**multi-mlp**	**SVM-rbf**	**LDA**
graph2vec	0.999 ± 0	0.999 ± 0	1 ± 0	0.83 ± 0.008*	0.789 ± 0.006*	0.822 ± 0.024*	0.644 ± 0.003*	0.498 ± 0.001*
MC-graph2vec	1 ± 0	1 ± 0	1 ± 0	0.864 ± 0.005*	0.998 ± 0.001	0.996 ± 0.004	0.761 ± 0.006*	0.574 ± 0.002*
MC-graph2vec (Index Labeled)	1 ± 0	1 ± 0	1 ± 0	0.993 ± 0.004*	0.998 ± 0.001	0.998 ± 0.001	1 ± 0	0.649 ± 0.007*
WGEVIA	1 ± 0	1 ± 0	1 ± 0	0.998 ± 0	0.998 ± 0.001	0.998 ± 0.001	1 ± 0	0.909 ± 0.002

**Table 6:** Results from evaluating the impact of modifications incorporated on top of graph2vec in our design of the WGEVIA algorithm—results for *nc* = 4 and μ*c* = 8.

	**Test accuracy (SIMU)**				**Test accuracy (REAL)**			
**Algorithm**	**Single-mlp**	**Multi-mlp**	**SVM-rbf**	**LDA**	**Single-mlp**	**Multi-mlp**	**SVM-rbf**	**LDA**
graph2vec	0.943 ± 0.018*	0.955 ± 0.014*	0.896 ± 0.027*	0.769 ± 0.035*	0.677 ± 0.011*	0.686 ± 0.008*	0.55 ± 0.008*	0.513 ± 0.007*
MC-graph2vec	0.961 ± 0.02*	0.963 ± 0.01*	0.924 ± 0.02*	0.798 ± 0.02*	0.733 ± 0.01*	0.783 ± 0.006*	0.713 ± 0.003*	0.561 ± 0.006*
MC-graph2vec (Index Labeled)	0.995 ± 0.006*	0.992 ± 0.008*	0.993 ± 0.005*	0.869 ± 0.02*	0.96 ± 0.002*	0.984 ± 0.002*	0.962 ± 0.001*	0.621 ± 0.007*
WGEVIA	1 ± 0	1 ± 0	1 ± 0	0.988 ± 0.009	0.986 ± 0.002	0.99 ± 0.002	0.986 ± 0.002	0.857 ± 0.005
	**Train accuracy (SIMU)**				**Train accuracy (REAL)**			
**Algorithm**	**Single-mlp**	**Multi-mlp**	**SVM-rbf**	**LDA**	**Single-mlp**	**Multi-mlp**	**SVM-rbf**	**LDA**
graph2vec	0.999 ± 0	0.999 ± 0	1 ± 0	0.83 ± 0.008*	0.789 ± 0.006*	0.822 ± 0.024*	0.644 ± 0.003*	0.498 ± 0.001*
MC-graph2vec	1 ± 0	1 ± 0	1 ± 0	0.787 ± 0.03*	0.977 ± 0.007*	0.993 ± 0.005*	0.734 ± 0.004*	0.531 ± 0.002*
MC-graph2vec (Index Labeled)	1 ± 0	1 ± 0	1 ± 0	0.877 ± 0.06*	0.996 ± 0.002	0.997 ± 0.002	0.997 ± 0	0.646 ± 0.005*
WGEVIA	1 ± 0	1 ± 0	1 ± 0	0.998 ± 0.009	0.996 ± 0.001	0.997 ± 0.001	0.997 ± 0.001	0.868 ± 0.004

In Tables 5, 6, we use “*” to mark a result if it indicates a significant difference (*p*-value < 0.05, pairwise *t*-test, two-tailed) between two designs. A given row *R* is only compared with rows below it, and methods in all rows below *R* can be regarded as reference methods for the method in the row *R*. For example, we compare graph2vec to MC-graph2vec with MC-graph2vec being the reference method.

### 3.8. Result Visualization

A distinguishing characteristic of WGEVIA is its multi-channel approach, where individual channels correspond to sets of unweighted graphs that are derived from the given microcircuit dataset *D* using thresholds that are applied to the edge weights. Each channel contains all of the unweighted graphs that result from a given threshold setting *T* (see Algorithm 3) when applied to *D*. A core part of WGEVIA is the iterative application of UGEVIA to each channel.

In this section, we show how visualizations can be used to gain intuitive insight into the contributions of different channels to the overall process in WGEVIA of deriving embedding vectors. For this purpose, we use t-Distributed Stochastic Neighbor Embedding (t-SNE) (van der Maaten and Hinton, 2008), which enables the visualization of high dimensional data by mapping each data point into a point in a two-dimensional space.

**Figure 4:**
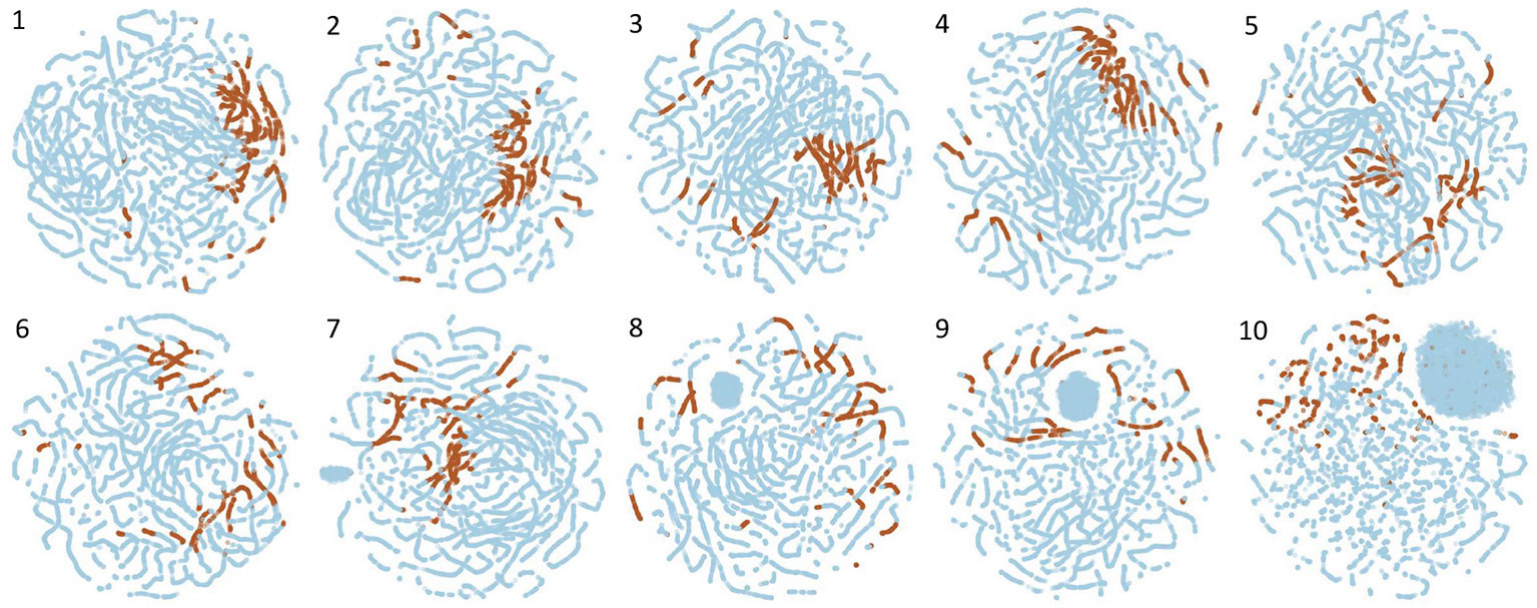
Visualizations derived using t-SNE for all channels when WGEVIA is applied to the REAL dataset with *nc* = 10 and μ*c* = 8.

Figure 4 shows visualizations derived using t-SNE for all channels when WGEVIA is applied to the REAL dataset with *nc* = 10 and μ*c* = 8. Plots are shown for each of the 10 channels, where higher channel indices (1–10) correspond to higher threshold values (*T* values) in Algorithm 3.

For a given plot in Figure 4, each point is derived from the embedding vector generated by UGEVIA for a specific unweighted graph within the channel associated with the plot. The t-SNE approach is used to project the μ*c*-dimensional embedding vector associated with each point into the two-dimensional space illustrated in the plot. A point is colored in blue if the corresponding unweighted graph is derived from a 0-labeled element of the REAL dataset, while brown-colored points correspond to 1-labeled elements.

Intuitively, we expect that a channel contributes more useful information to the output of WGEVIA if its embedding vectors are clustered together more tightly for the two different input labels (0 or 1). For example in Figure 4, we see that Channels 1 and 4 have most of the 1-labeled (brown) points clustered together, whereas the 1-labeled points are highly scattered in the plots for Channels 9 and 10. The application of the t-SNE visualization approach, as illustrated in Figure 4, therefore is useful in gaining insight into how different channels contribute to the output embedding vectors produced by WGEVIA, and the utility of the channels in helping a downstream classification task distinguish between different classes in the associated classification problem.

### 3.9. Runtime and Hyperparameter Tuning

**Figure 5:**
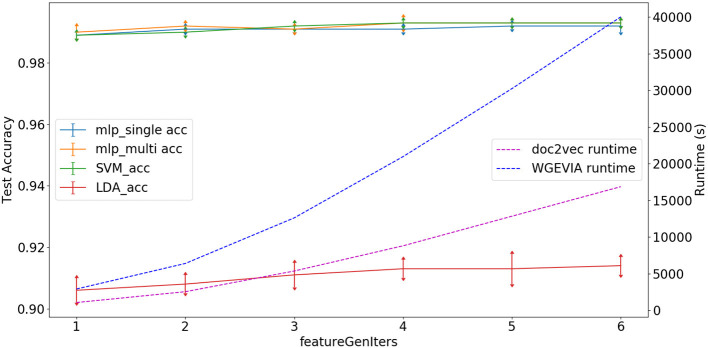
Test accuracy and runtime for different settings of the *featureGenIters* parameter when WGEVIA is applied to the REAL dataset with *nc* = 10 and μ*c* = 8.

As shown in Figure 5 and Table 7, we measured the runtimes *T*_*featureExtractor*_, *T*_*doc2vec*_, and *T*_*WGEVIA*_ of the UGEVIA feature extractor featureExtractor, doc2vec, and WGEVIA, respectively. The runtime measurements were performed with *featureGenIters* = [1, 2, 3, 4, 5, 6], *nc* = 10, and μ*c* = 8 on the REAL dataset (*n*_*G*_ = 17, 872, *k* = 420). We found that the product *n*_*G*_ × *T*_*featureExtractor*_ is far less (over 100 times less) than the runtime *T*_*doc2vec*_. As *featureGenIters* varies, *T*_*WGEVIA*_ remains almost linearly related to *T*_*doc2vec*_: *T*_*WGEVIA*_ ≈ α × *T*_*doc2vec*_, where α = 2.4 based on our experimental device and parallelism settings; we anticipate that the factor α arises mainly due to the overhead of executing UGEVIA in parallel. Detailed runtime complexity analysis of WGEVIA can be found in Appendix.

**Table 7:** Measured runtime of the UGEVIA featureExtractor, doc2vec, and WGEVIA with increasing values of the *featureGenIters* parameter.

**featureGenIters**	**1**	**2**	**3**	**4**	**5**	**6**
featureExtractor runtime (s)	0.00177	0.00368	0.00503	0.00683	0.00859	0.01011
doc2vec runtime(s)	1047.93	2528.76	5350.58	8791.51	12829.42	16861.66
WGEVIA runtime(s)	2894.96	6381.24	12600.51	20962.63	30241.33	40034.20

Based on Figure 5, the choice of *featureGenIters* does not significantly impact the embedding vector quality. This is because for the REAL dataset, smaller values of *featureGenIters* are sufficient to extract most of the information. For this reason, we are able to get high quality results with a setting of *featureGenIters* = 4 in our experiments.

**Figure 6:**
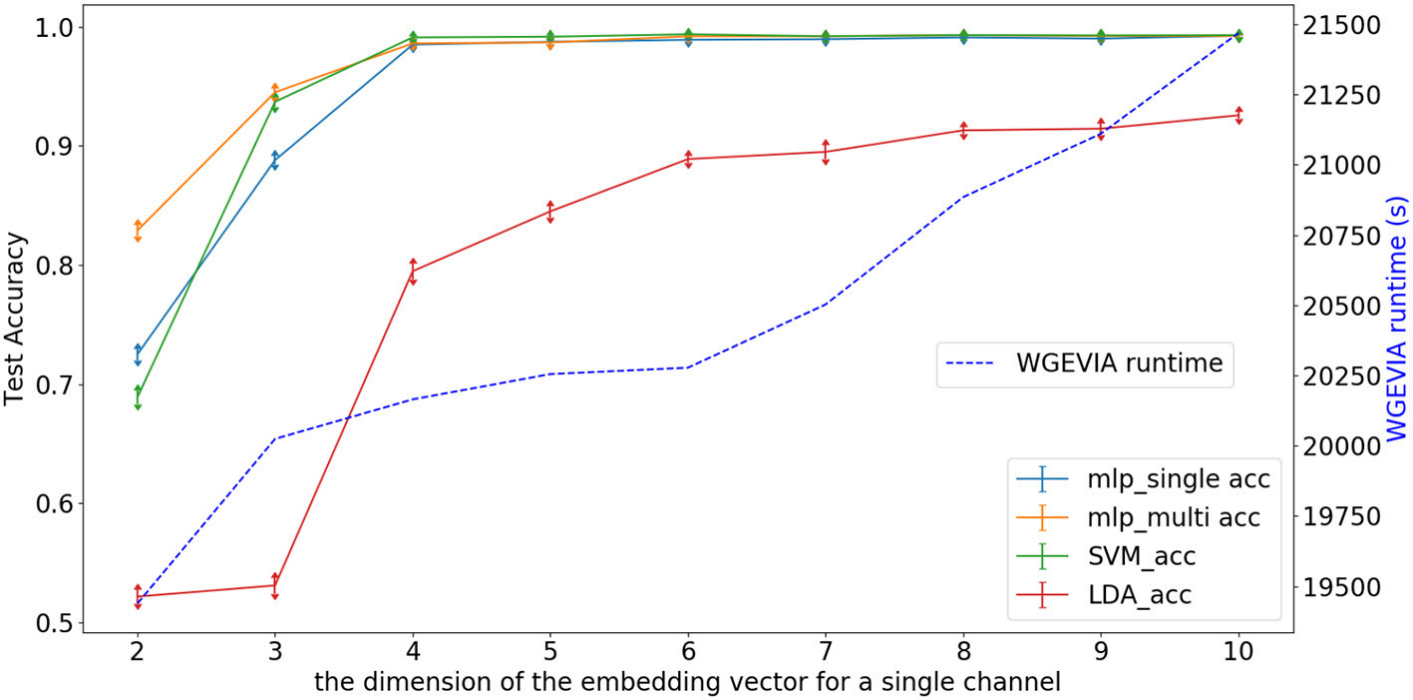
Test accuracy and runtime for different settings of the μ*c* parameter when WGEVIA is applied to the REAL dataset with *nc* = 10 and *featureGenIters* = 4.

**Figure 7:**
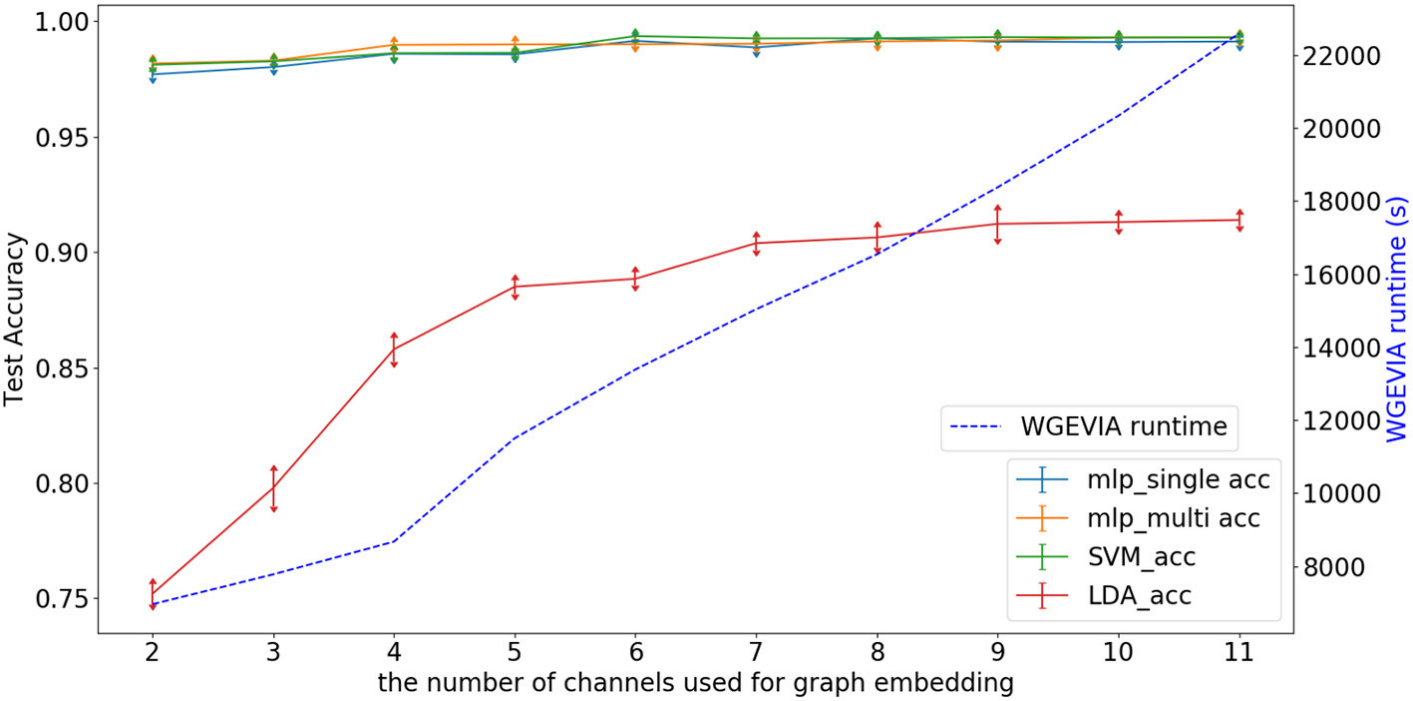
Test accuracy and runtime for different values of the *nc* parameter when WGEVIA is applied to the REAL dataset with μ*c* = 8 and *featureGenIters* = 4.

Figure 6 depicts experimental results with *nc* = 10, *featureGenIters* = 4, and μ*c* = [1, 2, …, 10]. In these results, we see that the classification accuracy increases as μ*c* increases and μ*c* = 8 is sufficient for WGEVIA to produce high quality results for the REAL dataset. By comparing with Figure 7, we see that the parameter μ*c* has less impact on the runtime of WGEVIA than *nc*.

**Figure 8:**
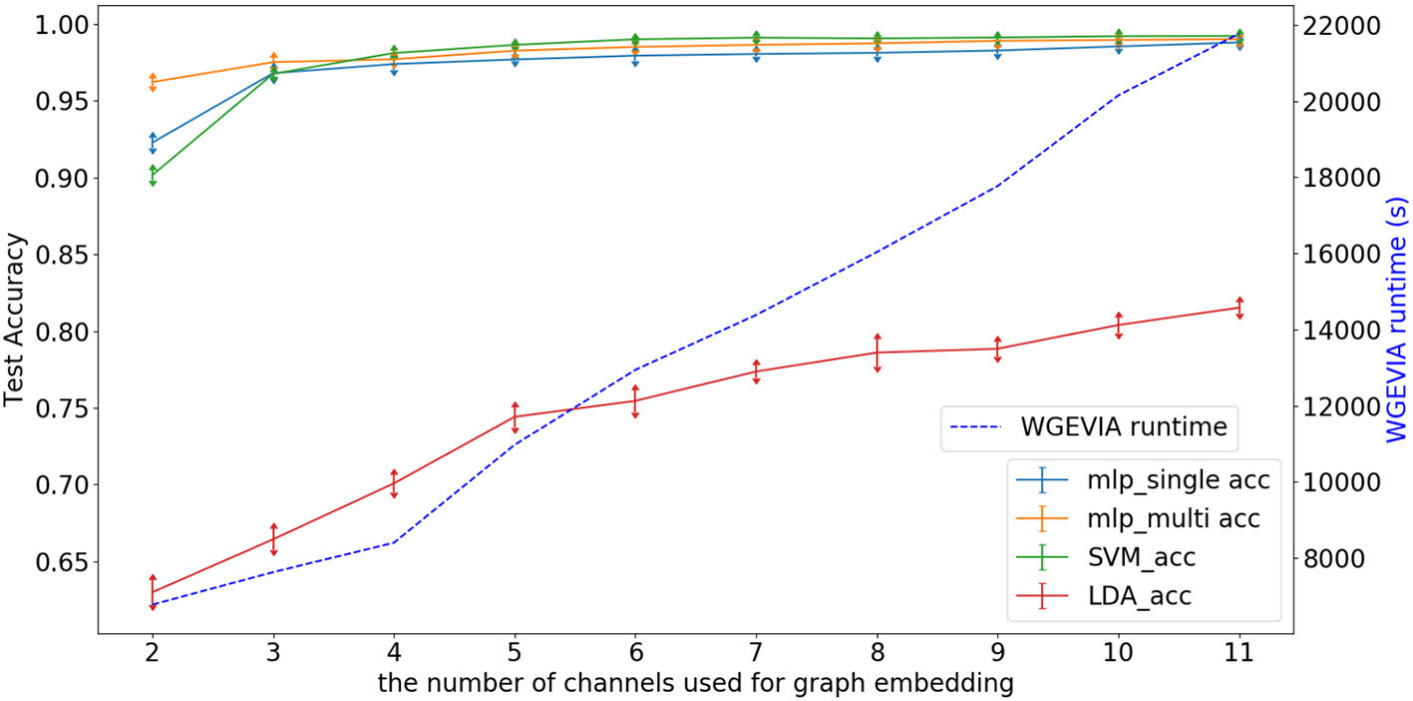
Test accuracy and runtime for different values of the *nc* parameter when WGEVIA is applied to the REAL dataset with μ*c* = 4 and *featureGenIters* = 4.

Figure 7 depicts experimental results with μ*c* = 8, *featureGenIters* = 4, and *nc* = [1, 2, …, 11]. In these results, the classification accuracy increases as *nc* increases. This demonstrates that the proposed multi-channel approach extracts more information when more channels are employed. The setting *nc* = 10 is sufficient for WGEVIA to produce high quality results for the REAL dataset. To further assess the impact of the *nc* parameter, we run experiments with μ*c* = 4 and the corresponding results are shown in Figure 8. Here, *nc* and μ*c* work together to impact on the embedding quality of WGEVIA. In these results, we see that with a smaller value of μ*c*, the impact of *nc* becomes more significant.

The runtime of the downstream classifiers depends on the number and dimension of the input embedding vectors and the structure of classifier models. In our experiments, the output embedding vector from WGEVIA has a dimension of *nc* × μ*c* = 80 since *nc* = 10 and μ*c* = 8. There are *n*_*G*_ = 17, 872 graphs in the REAL dataset and we use 90% of them for training. The MLP model has 128 nodes in the first hidden layer (*h*_1_ = 128), and 64 nodes in the second hidden layer (*h*_2_ = 64). The output layer has 2 nodes corresponding to the two binary classes (*o* = 2). The measured run time results for embedding vectors generated from the REAL dataset are shown in Table 8. All of the runtime results here are averaged over 10 runs.

**Table 8:** Runtime results for downstream classifiers.

	**Single-mlp**	**Multi-mlp**	**SVM**	**LDA**
Train time (s)	130.481	140.642	32.371	0.224
Inference time (ms)	0.0355	0.0361	2.1	0.000547

## 4. Conclusion and Discussion

In this paper, we have developed a novel algorithm, called Weighted Graph Embedding with Vertex Identity Awareness (WGEVIA), for embedding graph models of functional microcircuits. Distinguishing characteristics of WGEVIA that make it well-suited for functional microcircuit analysis include its ability to take graph weights and vertex identities into account. We also introduce a novel dataset, called the five vertices dataset, which helps to experiment with and evaluate how effective graph embedding algorithms are at taking vertex identities into account. WGEVIA introduces a novel concept of analyzing functional microcircuits in terms of well-defined collections of simplified (unweighted) graph models. These collections, called channels, can be visualized to gain insight into how different thresholds on between-neuron synchrony lead to different levels of behavior discrimination. Through extensive experiments using real and simulated microcircuit data, we demonstrate the effectiveness of the proposed new models and methods for graph embedding, and we show that the proposed methods outperform state-of-the-art graph embedding methods when applied to microcircuit data.

In this paper, we focus on undirected graphs. Edges in such undirected graphs quantify functional connectivity. Another kind of connectivity is effective connectivity. Two neurons are effectively connected if the firing of one neuron can trigger (or predict) the firing of another, without any assumption on how this effect is mediated (Feldt et al., 2011). A microcircuit describing effective connectivity is a directed graph. We may use the method in Chen et al. (2012) to detect effective connectivity. We plan to develop a graph embedding method for directed weighted graphs in our future work.

Current neural recording methods are limited in spatial coverage. For example, a typical calcium imaging study may observe several hundred neurons. Due to the limitation of the recording method, it is possible that the observed microcircuit is a subgraph of a local circuitry. With the advance of neural recording methods, which can observe 800,000–1,100,000 individual neurons across the dorsal surface of the neocortex (Kim et al., 2016), we will test our graph-embedding algorithm in these large-scale graphs in our future work.

The proposed method is a whole-graph embedding method and represents a whole-graph as a vector. In our future work, we plan to improve the model's interpretability by describing the relationship between individual graph vertices and the whole-graph embedding vector. Such analysis will shed light on which vertices contribute most to the whole-graph embedding vector.

Other interesting directions for future work include investigating extensions to WGEVIA for online graph embedding, for applicability beyond coherence-based microcircuit models, and for systematically optimizing the hyperparameters involved in WGEVIA.

## Data Availability Statement

 The original contributions presented in the study are publicly available. This data can be found here: The five vertices problem dataset is available for download from http://dspcad-www.iacs.umd.edu/bcnm/index.html. The data for the reward zone study is publicly available at https://datadryad.org/stash/dataset/doi:10.5061/dryad.rq560. The code is available under reasonable request.

## Ethics Statement

The animal study was reviewed and approved by The Columbia University Institutional Animal Care and Ethics Committee.

## Author Contributions

XW wrote the first draft of the manuscript and conducted experiments. SB and RC contributed to the conception and design of the study and review and revision of manuscript drafts. All authors contributed to the article and approved its submission.

## Conflict of Interest

The authors declare that the research was conducted in the absence of any commercial or financial relationships that could be construed as a potential conflict of interest.

## Appendix: Runtime Analysis

In this appendix, we analyze the time complexity of WGEVIA.

From Algorithm 3, the runtime *T*_*WGEVIA*_ of WGEVIA is *O*(*n*_*c*_×*T*_*UGEVIA*_), where *T*_*UGEVIA*_ is the runtime of UGEVIA. On a machine with sufficient resources, the channels can be processed in parallel so that the actual runtime does not necessarily scale linearly with *n*_*c*_; however, for the analysis in the remainder of this discussion, we do not take into account the potential for acceleration by exploiting parallelism.

From Algorithm 1, the runtime *T*_*UGEVIA*_ is *O*(*n*_*G*_ × *T*_*featureExtractor*_ + *T*_*doc2vec*_), where *n*_*G*_ is the set of input graphs, *T*_*featureExtractor*_ is the complexity of the UGEVIA feature extractor, and *T*_*doc2vec*_ is the complexity of doc2vec. The authors have been unable to find a complexity analysis of doc2vec in the literature so the expression *T*_*doc2vec*_ is left as as a “black box” in the remainder of our analysis in this appendix.

Finally, from Algorithm 2, the runtime *T*_*featureExtractor*_ can be expressed as *O*(*featureGenIters* × *k*^2^), where *k* is the number of vertices in a given input graph. The term *k*^2^ can be replaced by *k* for sparse graphs where the number of neighbors for a given vertex is bounded by a constant.

Putting the three pieces discussed above together yields the following time complexity expression for WGEVIA:

$$\begin{array}{l} O\left(n_{c}\times \left(\left(n_{G}\times featureGenIters\times k^{2}\right)+T_{doc2vec}\right)\right) \end{array}$$
